# Supplementary material for: Can scholarly pirate libraries bridge the knowledge access gap? An empirical study on the structural conditions of book piracy in global and European academia
Source: PLoS One. 2020 Dec 3;15(12):e0242509. doi: 10.1371/journal.pone.0242509 (PMC7714232; doi:10.1371/journal.pone.0242509)
Supplement: S1 File — (PDF) [file pone.0242509.s001.pdf]

Regional Eurostat Variables For Understanding Piracy Of Books

Daniel Antal, CFA

6/7/2020

The Data File

The `bookpiracy_2020-04-15.csv` is a csv export of the bookpiracy long-form data file found in `bookpiracy_2020-04-15.rda` in our GitHub Repo `bookpiracy_2020-04-15.rda`.

The `rda` file contains a wide format version of the data used and a detailed metadata table which is presented here in two tables for readability. The document identifier of the `csv` version of the data table `bookpiracy` is 10.21942/uva.12443465. The identifier of the metadata table, `bookpiracy_var_stat`, is 10.21942/uva.12443468. The `pdf` version of this long-form documentation is identified with 10.21942/uva.12443474

```
## Use your own path if you did not c
load (file = "../not_included/bookpiracy/bookpiracy_2020-04-15.rda")
write.csv ( bookpiracy, "bookpiracy_2020-04-15.csv",
            row.names = FALSE )
write.csv ( bookpiracy_var_stat,
            "bookpiracy_var_stat_2020-04-15.csv",
            row.names = FALSE )
```

Reference To Eurostat

The data file `bookpiracy_2020-04-15.rda` contains a summary table `bookpiracy_var_stat`. The first six colums give a summary of the original data source (and the next ones about our data processing steps, see after this one.)

Eurostat releases data in two forms: in simple data tables, or more complex products that can be filterred to simple tables. The following table shows the Eurostat product ID, the title of the statistic and the filters (i.e. `description`) applied to obtain the data programatically with `eurostat::get_eurostat`.

Eurostat ID	Description / Filter	
eurostat_code	title	description
lfst_r_lfe2emp	Employment by sex, age and NUTS 2 regions (1 000)	Total; 15 years or over [thousand]
tgs00096	Population on 1 January by NUTS 2 region	Total; total [number]
tgs00026	Disposable income of private households by NUTS 2 regions	Balance; disposable income, net [purchasing power standard (pps) per inhabitant]
tgs00003	Regional gross domestic product by NUTS 2 regions - million EUR	Regional gross domestic product by NUTS 2 regions - million EUR
tgs00004	Regional gross domestic product (million PPS) by NUTS 2 regions	Regional gross domestic product (million PPS) by NUTS 2 regions
tgs00005	Regional gross domestic product (PPS per inhabitant) by NUTS 2 regions	Regional gross domestic product (PPS per inhabitant) by NUTS 2 regions

(continued)

eurostat_code	title	description
tgs00109	Tertiary educational attainment, age group 25-64 by sex and NUTS 2 regions	Tertiary education (levels 5-8); from 25 to 64 years; total [percentage]
tgs00038	Human resources in science and technology (HRST) by NUTS 2 regions	Persons with tertiary education (isced) and/or employed in science and technology [percentage of active population]
tgs00042	Intramural R&D expenditure (GERD) by NUTS 2 regions	All sectors [percentage of gross domestic product (gdp)]
tgs00043	Researchers, all sectors by NUTS 2 regions	Researchers; total; all sectors [percentage of total employment - numerator in full-time equivalent (fte)]
isoc_r_iuse_i	Individuals who used the internet, frequency of use and activities	Frequency of internet access: daily [percentage of individuals]
isoc_r_iuse_i	Individuals who used the internet, frequency of use and activities	Internet use: internet banking [percentage of individuals]
isoc_r_iuse_i	Individuals who used the internet, frequency of use and activities	Internet use: participating in social networks (creating user profile, posting messages or other contributions to facebook, twitter, etc.) [percentage of individuals]
isoc_r_blt12_i	Individuals who ordered goods or services over the internet for private use	Last online purchase: in the 12 months [percentage of individuals]
rd_p_persreg	Total R&D personnel and researchers by sectors of performance, sex and NUTS 2 regions	Total; total; all sectors [full-time equivalent (fte)]
rd_p_persreg	Total R&D personnel and researchers by sectors of performance, sex and NUTS 2 regions	Total; total; all sectors [head count]
tgs00002	Total and land area by NUTS 2 region	Land area - total [square kilometre]

Variable IDs

The `eurostat_code` is an ambigous ID when filters are applied. Our custom ID `indicator` is simply a concatenation of the Eurostat ID and the filters applied with their respective metadata codes.

For example, `tgs00003_mio_eur` refers to the `tgs00003` data source applying the `mio_eur` (million euro) unit filter. The abbreviations are the Eurostat abbreviation, you can replicate our querry with them.

Variable Identification	
eurostat_code	indicator
lfst_r_lfe2emp	lfst_r_lfe2emp_t_y_ge15_ths
tgs00096	tgs00096_total_t_nr
tgs00026	tgs00026_bal_b6n_pps_hab
tgs00003	tgs00003_mio_eur
tgs00004	tgs00004_mio_pps
tgs00005	tgs00005_pps_hab
tgs00109	tgs00109_ed5-8_y25-64_t_pc
tgs00038	tgs00038_hrst_pc_act
tgs00042	tgs00042_total_pc_gdp
tgs00043	tgs00043_rse_t_total_pc_emp_fte
isoc_r_iuse_i	isoc_r_iuse_i_i_iday_pc_ind
isoc_r_iuse_i	isoc_r_iuse_i_i_iubk_pc_ind
isoc_r_iuse_i	isoc_r_iuse_i_i_iusnet_pc_ind
isoc_r_blt12_i	isoc_r_blt12_i_i_blt12_pc_ind
rd_p_persreg	rd_p_persreg_total_t_total_fte
rd_p_persreg	rd_p_persreg_total_t_total_hc
tgs00002	tgs00002_reg_area3_indicators

Unit Information

The unit information can be found in the `unit` (Eurostat abbreviation) and `unit_name` (Eurostat description) columns of the metadata table.

Custom ID	Unit Information	
indicator	unit	unit_name
lfst_r_lfe2emp_t_y_ge15_ths	THS	thousand
tgs00096_total_t_nr	NR	number
tgs00026_bal_b6n_pps_hab	PPS_HAB	purchasing power standard (pps) per inhabitant
tgs00003_mio_eur	MIO_EUR	million euro
tgs00004_mio_pps	MIO_PPS	million purchasing power standards (pps)
tgs00005_pps_hab	PPS_HAB	purchasing power standard (pps) per inhabitant
tgs00109_ed5-8_y25-64_t_pc	PC	percentage
tgs00038_hrst_pc_act	PC_ACT	percentage of active population
tgs00042_total_pc_gdp	PC_GDP	percentage of gross domestic product (gdp)
tgs00043_rse_t_total_pc_emp_fte	PC_EMP_FTE	percentage of total employment - numerator in full-time equivalent (fte)
isoc_r_iuse_i_i_iday_pc_ind	PC_IND	percentage of individuals
isoc_r_iuse_i_i_iubk_pc_ind	PC_IND	percentage of individuals
isoc_r_iuse_i_i_iusnet_pc_ind	PC_IND	percentage of individuals
isoc_r_blt12_i_i_blt12_pc_ind	PC_IND	percentage of individuals
rd_p_persreg_total_t_total_fte	FTE	full-time equivalent (fte)
rd_p_persreg_total_t_total_hc	HC	head count
tgs00002_reg_area3_indicators	KM2	square kilometre

Imputation Summary

Approximation on NUTS2 level

The imputation summary gives where we used the original data without modification, as downloaded programatically with the `eurostat::get_eurostat()` function from the Eurostat website.

The `Same Level` approximation methods were carried out on data aggregated on `NUTS2` level. When the data was not available for the year 2013, we tried first interpolated from the data of 2012 and 2014 with linear interpolation. If this was not possible, we tried to carry back the 2014 value (nocb = next observation carry back), then if this was not possible, we tried to carry forward the 2012 value (locf = last observation carry forward).

		Same NUTS2 Level		
indicator	actual	interpolated	nocb_actual	locf_actual
lfst_r_lfe2emp_t_y_ge15_ths	281	0	0	0
tgs00096_total_t_nr	271	0	10	0
tgs00026_bal_b6n_pps_hab	253	0	27	0
tgs00003_mio_eur	254	0	27	0
tgs00004_mio_pps	254	0	27	0
tgs00005_pps_hab	254	0	27	0
tgs00109_ed5-8_y25-64_t_pc	272	4	4	0
tgs00038_hrst_pc_act	281	0	0	0
tgs00042_total_pc_gdp	257	0	4	0
tgs00043_rse_t_total_pc_emp_fte	257	0	4	0
isoc_r_iuse_i_i_iday_pc_ind	135	0	19	0
isoc_r_iuse_i_i_iubk_pc_ind	132	0	19	0
isoc_r_iuse_i_i_iusnet_pc_ind	132	2	19	0
isoc_r_blt12_i_i_blt12_pc_ind	135	0	19	0
rd_p_persreg_total_t_total_fte	259	0	4	0
rd_p_persreg_total_t_total_hc	236	0	4	0
tgs00002_reg_area3_indicators	136	0	20	13

We have used the zoo package to approximate the regional time series. In the case of (linear) interpolation, the zoo package refers back to the basic `stats::approx()` function. We used `zoo` because of its better interface for programatic use (Zeileis and Grothendieck 2005, @R-zoo). [The following example uses a hypothetical data for limited, easier display.]

```
values <- example %>%
  eurostat::recode_to_nuts_2016()  %>%
  select ( values ) %>%
  unlist () %>%
  as.numeric()

cat(paste0 ("values = " , paste (values, collapse = ","),
            "\n" ))
```

```
## values = NA,NA,NA,51,52,55,NA,57,56,NA
```

```
approximated <- zoo::na.approx(object = values,
                               maxgap = 3, na.rm=FALSE )
cat(paste0 ( "approximated = " ,
             paste (approximated, collapse = ",") ),
    "\n")
```

```
## approximated = NA,NA,NA,51,52,55,56,57,56,NA
```

```r
nocb <- zoo::na.locf(object = approximated, fromLast=TRUE,
                     maxgap= Inf, na.rm=FALSE )
cat(paste0 ( "nocb = " , paste (nocb, collapse = ",") ),
    "\n")
```

```
## nocb = 51,51,51,51,52,55,56,57,56,NA
```

```r
locf <- zoo::na.locf(object = nocb, fromLast=FALSE,
                     maxgap= Inf, na.rm=FALSE )

cat(paste0 ( "locf = " ,
             paste (locf, collapse = ",")),
    "\n")
```

```
## locf = 51,51,51,51,52,55,56,57,56,56
```

Other Level/Source

The other level relates to the cases when we did not have data on the NUTS2 level, but we were able to find the data on NUTS1 level. Because the NUTS2 level data (in case of statistics computed from surveys) is an unknown weighted avarage of the NUTS2 regions constituting the NUTS1 region, this is a far more superior imputation strategy than the algorithms that are used for non-aggregated data. For example, if we have no data on Schwabia, standard algorithms would fill out the data for this region with a median or other value taken from all of Europe. Our method uses the data from Bavaria, and Schwabia is imputed with its own data and its neighboring Bavarian regions.

Because social and economic variables are generally strongly autocorrelated in space, and they are more homogeneous within a single nation state and language area, using the hierarchical territorial aggregation structure of the NUTS statistics we receive a far better approximation than if we treated the missing observations independent from their (geographical) neighbors.

		Other NUTS Level or Source			
indicator	actual	NUTS1 actual	NUTS1 nocb	from tgs00002	national source
lfst_r_lfe2emp_t_y_g	281	0	0	0	0
tgs00096_total_t_nr	271	0	0	0	0
tgs00026_bal_b6n_pp	253	0	0	0	0
tgs00003_mio_eur	254	0	0	0	0
tgs00004_mio_pps	254	0	0	0	0
tgs00005_pps_hab	254	0	0	0	0
tgs00109_ed5-8_y25-64_t_pc	272	0	0	0	0
tgs00038_hrst_pc_act	281	0	0	0	0
tgs00042_total_pc_gd	257	0	0	0	0
tgs00043_rse_t_total_pc_emp_ft	257	0	0	0	0
isoc_r_iuse_i_i_iday_	135	114	1	0	0
isoc_r_iuse_i_i_iubk_pc_ind	132	117	1	0	0
isoc_r_iuse_i_i_iusne	132	115	2	0	0
isoc_r_blt12_i_i_blt12_pc_ind	135	114	1	0	0
rd_p_persreg_total_t	259	0	0	0	0
rd_p_persreg_total_t_total_hc	236	0	0	0	0
tgs00002_reg_area3_i	136	0	0	82	5

Again, if the NUTS1 data was not avaialble for the year 2013, we tried to carry back the 2014 observation, or carry forward the 2012 observation, and then project it to the constitutent NUTS2 region. Much of this methodology was released on CRAN as a result of this work in the package regions (see functions: impute_down and impute_down_nuts.)

Interestingly, the land area used for normalization was not available for all NUTS2 units. The reason for this is that only those NUTS regions report separate total and land area, where there are large water bodies present within the boundaries (such as marine bays or lakes.) We used the total NUTS2 area when the land area was not separately given, and we had to find this information in a few cases from national sources.

In our view, this is not even imputation, but finding the relevant information under a different metadata header.

Much of the program code that we used to create these datasets find their way into the R package `regions`, which was released after peer-review on CRAN. Our datasets were created with earlier versions of the code. The algorithm of imputation using the geographical structure of the NUTS typology can be found in impute_down_nuts() for Eurostat data and in a more general form impute_down().

Regional Identifier

Our article was almost finished more than a year earlier, but our reproducible program code stopped working at one point. The reason was that we used a programmatic access to Eurostat's regional database, but Eurostat changed the metadata structure and library of geographical coding. Because our data relates to the period of the `NUTS2013` geographical boundary definitions (i.e. regional boundaries as defined in the 2013 edition of NUTS), but Eurostat stopped making data available in this typology, we had to convert back Eurostat's data from the currently used `NUTS2016` typology to `NUTS2013`. This was not always possible, however, new data became available in the new typology, so in terms of data coverage, we rather gained than lost useful data.

The conversion is, however, anything but straightforward. Our initial program code was about 700 lines, and in a more robust form found their way into the rOpenGov package for programmatic access of Eurostat data, eurostat. Later, it formed the basis of a brand-new R package regions on CRAN that was first released after peer review on 4 June 2020 (Lahti et al. 2017, 2020; Antal 2020). Our dataset was created with earlier versions of the code, which went on countless revisions to correctly handle more and more exceptions.

Here we only briefly introduce the problems we faced when creating this dataset. The website of the new regions package gives a far more comprehensive overview on the problems of joining sub-national data from different sources. The problem is, in short, that while national boundaries are relatively stable, within states, boundaries of provinces, regions, counties and other sub-national divisions change several dozen times every few years in Europe. Data from different sources almost never uses the same internal boundaries. Correctly identifying the territorial unit that was used for computing a statistic is often rather challenging.

Let's take a purely hypothetical but easy to understand example for data available in `Limousin`, France. In this hypothetical data frame, data is coded according to the old region codes till 2016, and in 2017 only for the region larger `Aquitaine-Limousin-Poitou-Charentes`.

geo	time	code13	code16	change	resolution	nuts_level	name
FR63	2010-01-01	FR63	FRI1	recoded	FR63=FRI2	2	Limousin
FR63	2011-01-01	FR63	FRI1	recoded	FR63=FRI2	2	Limousin
FR63	2012-01-01	FR63	FRI1	recoded	FR63=FRI2	2	Limousin
FR63	2013-01-01	FR63	FRI1	recoded	FR63=FRI2	2	Limousin
FR63	2014-01-01	FR63	FRI1	recoded	FR63=FRI2	2	Limousin
FR63	2015-01-01	FR63	FRI1	recoded	FR63=FRI2	2	Limousin
FR63	2016-01-01	FR63	FRI1	recoded	FR63=FRI2	2	Limousin
FRI	2017-01-01	FR6	FRI	recoded	FR6=FRI	1	AQUITAINE-LIMOUSIN-POITOU-CHARENTES
FRI2	2018-01-01	FR63	FRI1	recoded	FR63=FRI2	2	Limousin
FRI2	2019-01-01	FR63	FRI1	recoded	FR63=FRI2	2	Limousin

In this case, the Limousin region's boundaries did not change, but Limousin got a new `NUTS2` code, `FRI`. The data for the year 2013 is available in the dataset, but under the earlier code `FR63`.

geo	time	code13	code16	change	resolution	nuts_level	name
FRI1	2010-01-01	FR63	FRI1	recoded	FR63=FRI2	2	Limousin
FRI1	2011-01-01	FR63	FRI1	recoded	FR63=FRI2	2	Limousin
FRI1	2012-01-01	FR63	FRI1	recoded	FR63=FRI2	2	Limousin
FRI1	2013-01-01	FR63	FRI1	recoded	FR63=FRI2	2	Limousin
FRI1	2014-01-01	FR63	FRI1	recoded	FR63=FRI2	2	Limousin
FRI1	2015-01-01	FR63	FRI1	recoded	FR63=FRI2	2	Limousin
FRI1	2016-01-01	FR63	FRI1	recoded	FR63=FRI2	2	Limousin
FRI	2017-01-01	FR6	FRI	recoded	FR6=FRI	1	AQUITAINE-LIMOUSIN-POITOU-CHARENTES
FRI1	2018-01-01	FR63	FRI1	recoded	FR63=FRI2	2	Limousin
FRI1	2019-01-01	FR63	FRI1	recoded	FR63=FRI2	2	Limousin

While the year 2017 is of no interest to our models, for simpler demonstration we remain with this example. In this case, we do not have actual data for `Limousine` (`FRI1`), but we have data for the `NUTS1` level larger region `Aquitaine-Limousin-Poitou-Charentes`.

In reality, this problem is unlikely to present itself for one region. In the case of some statistics based on Eurobarometer and other relatively small sample surveys, for the larger member states the statistics is only calculated at `NUTS1` (larger region) level. If the larger region statistic is an unknown weighted averages of the smaller constituent `NUTS2` regions, we can safely impute the larger regions's value to the smaller regions.

In this hypothetical example, the value of `Aquitaine-Limousin-Poitou-Charentes` is in fact an average value (with unknown weighting) of `Aquitaine`, `Limousin`, `Poitou` and `Charentes`.

geo	time	code13	code16	nuts_level	name	values	method
FRI1	2010-01-01	FR63	FRI1	2	Limousine	NA	missing
FRI1	2011-01-01	FR63	FRI1	2	Limousine	NA	missing
FRI1	2012-01-01	FR63	FRI1	2	Limousine	NA	missing
FRI1	2013-01-01	FR63	FRI1	2	Limousine	51	actual
FRI1	2014-01-01	FR63	FRI1	2	Limousine	52	actual
FRI1	2015-01-01	FR63	FRI1	2	Limousine	55	actual
FRI1	2016-01-01	FR63	FRI1	2	Limousine	NA	missing
FRI1	2017-01-01	FR63	FRI1	1	Limousine	57	imputed from NUTS1 actual
FRI1	2018-01-01	FR63	FRI1	2	Limousine	56	actual
FRI1	2019-01-01	FR63	FRI1	2	Limousine	NA	actual

To summarize, these are the actual changes in the hypothetical example:

geo	time	code13	code16	name	values	method
FRI1	2010-01-01	FR63	FRI1	Limousine	51	nocb
FRI1	2011-01-01	FR63	FRI1	Limousine	51	nocb
FRI1	2012-01-01	FR63	FRI1	Limousine	51	nocb
FRI1	2013-01-01	FR63	FRI1	Limousine	51	actual
FRI1	2014-01-01	FR63	FRI1	Limousine	52	actual
FRI1	2015-01-01	FR63	FRI1	Limousine	55	actual
FRI1	2016-01-01	FR63	FRI1	Limousine	56	imputed from NUTS1 actual
FRI1	2017-01-01	FR63	FRI1	Limousine	57	actual
FRI1	2018-01-01	FR63	FRI1	Limousine	56	actual
FRI1	2019-01-01	FR63	FRI1	Limousine	56	locf

R Software References

Antal, Daniel. 2020. *Regions: Processing Regional Statistics*. https://regions.danielantal.eu/.

Lahti, Leo, Janne Huovari, Markus Kainu, and Przemyslaw Biecek. 2017. "Eurostat R Package." *R Journal*. https://journal.r-project.org/archive/2017/RJ-2017-019/index.html.

———. 2020. *Eurostat: Tools for Eurostat Open Data*. https://CRAN.R-project.org/package=eurostat.

Zeileis, Achim, and Gabor Grothendieck. 2005. "Zoo: S3 Infrastructure for Regular and Irregular Time Series." *Journal of Statistical Software* 14 (6): 1–27. https://doi.org/10.18637/jss.v014.i06.

Zeileis, Achim, Gabor Grothendieck, and Jeffrey A. Ryan. 2020. *Zoo: S3 Infrastructure for Regular and Irregular Time Series (Z's Ordered Observations)*. https://CRAN.R-project.org/package=zoo.
