# Supplementary material for: Can scholarly pirate libraries bridge the knowledge access gap? An empirical study on the structural conditions of book piracy in global and European academia
Source: PLoS One. 2020 Dec 3;15(12):e0242509. doi: 10.1371/journal.pone.0242509 (PMC7714232; doi:10.1371/journal.pone.0242509)
Supplement: S1 Table — (PDF) [file pone.0242509.s002.pdf]

	skim_type	skim_variable	n_missing	complete_rate	numeric.mean	numeric.sd	numeric.p0	numeric.p25	numeric.p50	numeric.p75	numeric.p100
1	numeric	Downloads	0	1.0000000	6.252349e+04	1.735601e+05	1.00000	467.00000	7.442000e+03	4.910400e+04	1.683353e+06
2	numeric	Population	15	0.9333333	3.480721e+07	1.354592e+08	17665.00000	961505.75000	6.714955e+06	2.368659e+07	1.371220e+09
3	numeric	GDP	33	0.8533333	1.966143e+04	2.053627e+04	841.42209	4265.67274	1.231611e+04	2.705457e+04	1.166232e+05
4	numeric	Internet penetration	21	0.9066667	4.096251e+06	2.119797e+07	117.00000	16862.00000	1.881255e+05	1.789139e+06	2.770460e+08
5	numeric	Spending on R&D	89	0.6044444	8.110186e−01	9.152203e−01	0.01497	0.17673	4.433900e−01	1.055902e+00	4.255630e+00
6	numeric	Literacy rate	71	0.6844444	8.279893e+01	2.040405e+01	26.00299	72.43376	9.286848e+01	9.817273e+01	9.999819e+01
7	numeric	Expenditure on tertiary ed. per student	113	0.4977778	6.221563e+03	6.018732e+03	148.00000	1866.00000	4.199500e+03	7.895000e+03	2.480500e+04
8	numeric	H − Index	7	0.9688889	1.791193e+02	2.492286e+02	1.00000	40.50000	8.350000e+01	1.877500e+02	1.965000e+03
