# Supplementary material for: Can scholarly pirate libraries bridge the knowledge access gap? An empirical study on the structural conditions of book piracy in global and European academia
Source: PLoS One. 2020 Dec 3;15(12):e0242509. doi: 10.1371/journal.pone.0242509 (PMC7714232; doi:10.1371/journal.pone.0242509)
Supplement: S2 Table — (PDF) [file pone.0242509.s003.pdf]

	names	Model 1	Model 2	Model 3
1		Model 1	Model 2	Model 3
2	(Intercept)	0.621121110037844	0.573066439572682	0.903188698275772
3		(2.1132612051991)	(2.11727496547961)	(2.4913320060074)
4	log(gdp_pps)	0.955126254008121 ***	0.959098557184983 ***	0.963732681153714 ***
5		(0.0632768251831421)	(0.0640742589642845)	(0.0702790551866092)
6	researcher_employment_pct	0.31815049303284 ***	0.327162738184005 ***	0.406195748307072 ***
7		(0.0863298463024823)	(0.0890344766435439)	(0.101020168398562)
8	log(disposable_income)	−0.10286601233766	−0.0997598068258344	−0.144832003657147
9		(0.246038584087386)	(0.246169546592807)	(0.292788556608312)
10	edu_attainment_total	0.0147511373412976 *	0.0138489819654799	0.00476863460551328
11		(0.00743910231457829)	(0.00776968174391031)	(0.00897387270812049)
12	internet_use_banking_pc	−0.0195794874023834 ***	−0.0189299949909371 ***	
13		(0.00346210684298085)	(0.00380776198876701)	
14	gerd		−0.0190133190881826	−0.0679065142371642
15			(0.0466327558351086)	(0.0533571306807522)
16	internet_purchases_last_year_pc			−0.00961346458836273 *
17				(0.00414071144968771)
18	nobs	265	265	265
19	null.deviance	7192467.38245742	7192467.38245742	7192467.38245742
20	df.null	264	264	264
21	logLik	NA	NA	NA
22	AIC	NA	NA	NA
23	BIC	NA	NA	NA
24	deviance	1992084.22079746	1990052.04205723	2228815.78269504
25	df.residual	259	258	258
26	pseudo.r.squared	1	1	1
27	pseudo.r.squared.mcfadden	0.722746298399355	0.723028729446555	0.689845480371716
28	*** p < 0.001; ** p < 0.01; * p < 0.05.			
