# Supplementary material for: Can scholarly pirate libraries bridge the knowledge access gap? An empirical study on the structural conditions of book piracy in global and European academia
Source: PLoS One. 2020 Dec 3;15(12):e0242509. doi: 10.1371/journal.pone.0242509 (PMC7714232; doi:10.1371/journal.pone.0242509)
Supplement: Attachment — Submitted filename: Reviewer Recommendation and Comments for Manuscript Number PONE-D-20-18408.docx [file pone.0242509.s004.docx]

The paper “Open access is not a panacea, even if it’s radical – an empirical study on the role of shadow libraries in closing the inequality of knowledge access” investigates on the economic and social factors behind the usage of shadow libraries, and provides interesting analysis in the global and European models. The publishers' subscription fee increases the financial burden of institutions and researchers. Shadow library was created to deal with complex institutional, political, financial, and other issues. However, academic piracy is worth studying, so this study is indeed a relevant and timely topic. This article is well organized and draws conclusions and arguments on the data analysis. I only have a few minor comments for the authors to consider, as to further improve the article:

1. In the “Data Overview and Descriptive Statistics” section, the author obtained many data sources, which provided the source and explanation of variables for the following model. It is important to show a table with the descriptive statistical information of variables in the model. This table can show the information of variables more intuitively and enhance readability.

2. In the construction of Global/European model, independent variables should use literature to support, especially for the authors to justify their research design (why some indicators were considered, but not the others)

3. In the Global Model, the authors write " Since our dependent variable is count data, the use of Poisson regression is justified. On the other hand, a negative binomial distribution did not suit this problem, therefore we omitted that approach " When the dependent variable is count data, Poisson regression and negative binomial regression are optional. Although Poisson regression is usually used, it needs explanation why negative binomial distribution is not suitable for this problem. If possible, the author should declare the reason for selecting regression methods, such as the relationship between expectation and variance.

4. There are three identical tables 3. I don't understand why three identical tables should be here.

5. In the Random forest models, I believe that the random forest model selected by the author has performed well in this study, but other model selection methods should also be considered. If possible, authors can add the comparison between other methods and the random forest model, which can better explain why the random forest method is used instead of others.
