# Supplementary material for: Can scholarly pirate libraries bridge the knowledge access gap? An empirical study on the structural conditions of book piracy in global and European academia
Source: PLoS One. 2020 Dec 3;15(12):e0242509. doi: 10.1371/journal.pone.0242509 (PMC7714232; doi:10.1371/journal.pone.0242509)
Supplement: Attachment — Submitted filename: response_to_reviewers.docx [file pone.0242509.s005.docx]

Rebuttal letter, manuscript ID#PONE-D-20-18408

Dear Reviewers, editors,

First of all we would like to thank you for the useful reviews, and comments. Hopefully we addressed each point fully. This letter is thus less of a rebuttal, than a confirmation of us addressing each point as requested.

1. **Please ensure that your manuscript meets PLOS ONE's style requirements, including those for file naming.** We updated the document to conform with the requirements:
   - changed the style of headings
   - updated the formatting of references
   - table titles have been moved on top of the tables
   - figures have been renamed to Fig.
   - tables have been converted to proper tables.

2. **Please consider changing the title so as to meet our title format requirement.**

We changed the title to Can scholarly pirate libraries bridge the knowledge access gap? An empirical study on the structural conditions of book piracy in global and European academia.

2. **We note that one or more of the authors are employed by a commercial company.**

**We would like to make a correction and requested statements for author Daniel Antal.**

We would like to state that the Daniel Antal is an independent researcher. The affiliation information provided was confusing, and we would like to change it. CEEMID is not a commercial entity, as you have suggested in your remarks, but a trademark registered in the Dutch Chamber of Commerce to the sole proprietorship of Daniel Antal and a name of a collaborative project to pool open and other research data. This name and trademark was used as AD’s affiliation because the article uses some of the code and (open) data that CEEMID also uses.

Daniel Antal is a sole proprietor under Dutch law, and he mainly provides data analysis. There was no employer, client or other funder that played any role in the study design, data collection and analysis, decision to publish, or preparation of the manuscript. Furthermore, he has no whatsoever conflicting interest, personal, professional or financial that is related anyhow to this publication, and his other activities as a sole proprietor do not alter our adherence to PLOS ONE policies on sharing data and materials. The only funding that Daniel Antal received for this work was paid from the identified funding source of this project, so no additional funding information is applicable.

**Therefore, we believe that correct affiliation in this case is “independent researcher”.**

4. **We note that you have stated that you will provide repository information for your data at acceptance.**

We added the DOIs for both the data and the code to the paper.

Code and data repository: <https://zenodo.org/record/4012352>; DOI: 10.5281/ZENODO.4012352

Raw Data repository: <https://uvaauas.figshare.com/projects/Shadow_Libraries/80837>; DOI: 10.21942/uva.12330959

**5. We note that Figure 2 and 4 in your submission contain map images which may be copyrighted.**

Both figures were created using open source software leaflet, and open access data by Openstreetmap. In both figures the copyright information is visible. Maps based on Openstreetmap data have been published before in PLOS ONE (for example: <https://journals.plos.org/plosone/article/figure?id=10.1371/journal.pone.0165331.g001> and <https://journals.plos.org/plosone/article/figures?id=10.1371/journal.pone.0209641>) We updated the description to include the appropriate copyright information.

6. **Please ensure that you refer to Figures 2, 4 and 5 in your text as, if accepted, production will need this reference to link the reader to the figures.** DONE

7. **Please ensure that you refer to Tables 5, 7 and 10 in your text; if accepted, production will need this reference to link the reader to the Tables.** DONE

8. P**lease include captions for your Supporting Information files at the end of your manuscript, and update any in-text citations to match accordingly.** Done.

Reviewers’ comments

**1. In the “Data Overview and Descriptive Statistics” section, the author obtained many data sources, which provided the source and explanation of variables for the following model. It is important to show a table with the descriptive statistical information of variables in the model. This table can show the information of variables more intuitively and enhance readability.**
For readability we included this table as supplementary material S2 Table.

**2. In the construction of Global/European model, independent variables should use literature to support, especially for the authors to justify their research design (why some indicators were considered, but not the others)**
We added extra language to the Data overview and descriptive statistics section to address this.

**3. In the Global Model, the authors write " Since our dependent variable is count data, the use of Poisson regression is justified. On the other hand, a negative binomial distribution did not suit this problem, therefore we omitted that approach " When the dependent variable is count data, Poisson regression and negative binomial regression are optional. Although Poisson regression is usually used, it needs explanation why negative binomial distribution is not suitable for this problem. If possible, the author should declare the reason for selecting regression methods, such as the relationship between expectation and variance.**
We added extra language and reference to justify our use of Poisson regression as opposed to neg binomial.

**4. There are three identical tables 3. I don't understand why three identical tables should be here.**

We could not find three identical tables in the submitted manuscript.

**5. In the Random forest models, I believe that the random forest model selected by the author has performed well in this study, but other model selection methods should also be considered. If possible, authors can add the comparison between other methods and the random forest model, which can better explain why the random forest method is used instead of others.**

We added extra language to better explain other methods we used, and the role of random forest in the study.

Reviewer #2:

1. **The names of variables should be addressed clearly, such as “dl_per_pop_round”, “dl_per_pop”, “pop_per_mil”, “eb_is_visit_public_library” , “eb_limited_library_supply”, et al.** Corrected.

**2. What is the statistical meaning of value in parentheses below the coefficient in each model?** Standard errors are in the parentheses. Added this info to the text.

3. **Table 3 appears three times, and table 6 appears twice. Figure 6 is not shown in the manuscript. Please check the layout and details carefully.**
As above, in the submitted manuscript we could not find these errors.

**4. In page 15 and 16, authors claimed the model 7 can “explain ~72% of the variance”, “effect of disposable income is positive and significant at a 95% level” in model 9, and “the download per researcher models have a worse fit than the per capita models”. How statistics support these statements? Could the certifiable statistics be available?**
Added language. All statistics are available in the code repository.

**5. It seems not very clear to understand how the results of Random forest models connect with other models or support the research conclusion. Would the results of Random forest models be available and explained more specifically and clearly?**

Added extra language to explain better the use of random forest models.
